# Unraveling the surface marker signature of cell-derived vesicles via proteome analysis and nanoparticle flow cytometry

**DOI:** 10.1038/s41598-023-50279-x

**Published:** 2024-01-02

**Authors:** Hui-Chong Lau, Ilaria Passalacqua, Jik-Han Jung, Yerim Kwon, Davide Zocco, Sung-Soo Park, Seung Wook Oh

**Affiliations:** 1 BioDrone Research Institute, MDimune Inc., Seoul, South Korea; 2 Lonza Siena., Siena, Italy; 3 BioDrone Therapeutics Inc., Seattle, USA

**Keywords:** Nanobiotechnology, Nanobiotechnology

## Abstract

The cell-derived vesicles (CDVs) obtained using a proprietary extrusion process are the foundation of BioDrone platform technology. With superior productivity and versatility, this technology has garnered increasing attention in broad applications, particularly as a drug delivery vehicle. Previously, we showed that CDVs exhibited varying levels of expression for tetraspanin and organelle membrane markers while revealing no discernible differences in physical characteristics compared to naturally produced extracellular vesicles (EVs). To further understand and utilize the therapeutic potentials of CDVs, a more comprehensive study of membrane protein profiles is necessary. In addition, it is crucial to validate that the CDVs produced from extrusion are indeed intact lipid vesicles rather than other impurities. Here, we produced multiple batches of CDVs and EVs from HEK293 cells. CDVs and EVs were subjected to the same purification processes for subsequent proteome and particle analyses. The proteome analyses revealed unique proteome signatures between CDVs, EVs, and parental cells. Extensive proteome analyses identified the nine most prominent membrane markers that are abundant in CDVs compared to cells and EVs. Subsequent western blotting and nanoparticle flow cytometry analyses confirmed that CD63, lysosome-associated membrane glycoprotein 1 (LAMP1), and nicastrin (NCSTN) are highly enriched in CDVs, whereas CD81, CD9, and prostaglandin F2 receptor negative regulator (PTGFRN) are more abundant in EVs. This highlights the unique membrane composition and marker signature of CDVs that are distinct from EVs. Lastly, we demonstrated that more than 90% of the CDVs are genuine lipid vesicles by combining two different classes of vesicle labeling dyes and detergents to disrupt lipid membranes. This indicates that our proprietary extrusion technology is highly compatible with other well-characterized EV production methods. The robust CDV markers identified in this study will also facilitate the engineering of CDVs to achieve enhanced therapeutic effects or tissue-selective cargo delivery.

## Introduction

The global pandemic has brought lipid nanoparticles (LNPs) to the forefront as a clinic option to choose for mRNA delivery. However, their safety issues, such as immunogenicity and long-term safety, remain a subject of further investigation^1,2^. For instance, in the results of clinical trials for both BioNTech/Pfizer and Moderna vaccines, a significant percentage of subjects presented local and systemic adverse events^3,4^. In rodents, LNPs administered via three different routes (intradermal, intramuscular, and intranasal) induced activation of diverse inflammatory pathways that may be the underlying mechanism of the side effects observed in humans^5^. While the mild adjuvant activity of LNPs can be a useful feature as a vaccine platform^6^, it would greatly limit the use of LNPs as a gene delivery vehicle for broad clinical applications, particularly those requiring systemic injection of high doses.

While challenges related to LNPs’ safety persist, naturally occurring nanoparticles, such as extracellular vesicles (EVs), have garnered tremendous attention owing to their superior biocompatibility^7,8^. As highly conserved molecular shuttles in nature, EVs play a significant role in intercellular communication in various physiological processes by delivering diverse biomolecular cargo to recipient cells^9^. A distinctive feature of EVs is their complex surface repertoire, which appears important for efficient cellular uptake and tissue-specific targeting^8,9^. Excellent safety profiles of EVs will help overcome the toxicity of LNPs^9,10^. Recently completed clinical trials on EV-based therapeutics have reported that EVs are well tolerated in humans^11–13^. Studies have also shown comparable levels of therapeutic delivery between EVs and LNPs^14–16^. However, the low productivity of EVs is currently a significant challenge that may prevent broad use in clinical applications^17,18^.

Cell-derived vesicles (CDVs), with their superior productivity, have emerged as promising drug delivery vehicles to address such limitations of EVs. CDVs are similar to EVs in morphology and many other physicochemical properties. However, CDVs are produced at a much higher yield, up to 100 times greater than EVs^19,20^. The overall production time for CDVs is significantly shorter than that of EVs. For instance, the extrusion process to obtain CDVs takes less than 1% of the total time required for EV production, which typically requires 24–48 h, even on a research scale. Thus, by improving both output per unit cells and process time, the CDV technology will minimize the need for extensive cell culture and enable fast and cost-effective nanovesicle production. Moreover, CDV production can be readily scaled for commercial use, while EVs isolated from UC and commercial kits may not^7^. Our previous study has successfully demonstrated the transfer of the research scale manufacturing process to an SOP-guided GMP-compliant process with high batch-to-batch consistency^21^. GMP-compliant manufacturing of CDVs will facilitate future therapeutic development based on the CDV technology.

Many studies have demonstrated the potential of CDVs in diverse areas, including cancer, inflammatory disorders, and regenerative medicine. For example, CDVs have been shown to have anti-cancer effects on various cancer animal models^22,23^. They have also been shown to reduce inflammation and promote tissue regeneration when derived from potent stem cells or immune cells^24–26^. In addition to their therapeutic potential, CDVs are also excellent drug carriers with the ability to target specific cells or tissues, which can drastically improve drug efficacy^20,27^. Importantly, CDVs exhibit excellent safety profiles in vitro and in vivo^28^. Taken together, their superior productivity, therapeutic potential, and excellent safety profiles make CDVs a promising new option for delivering therapeutic agents.

Our previous study highlighted the similarities and differences between umbilical cord mesenchymal stem cell (UCMSC)-derived CDVs and -EVs^21^. Notably, CD9 and CD81, which are well-established EV markers, are found in lower abundance in CDVs compared to EVs. On the contrary, CD63 and subcellular organelle markers such as lysosome-associated membrane glycoprotein 1 (LAMP1) are more prominent in CDVs. Thus, to better understand the surface marker signatures of CDVs and consequently foster the development of drug delivery platforms based on CDVs, detailed analyses at a molecular level are necessary for each cell type. Finally, the precise assessment of the purity of CDVs produced through the extrusion technology, relative to naturally secreted EVs, will add confidence to this relatively novel nanovesicle technology.

Here, we present a comprehensive approach integrating molecular profiling and purity assessment for CDVs. The identification of enriched CDV proteins offers extensive knowledge of the CDV-specific markers, which in turn contribute to targeted delivery or more efficient cargo loading by leveraging such specific protein markers in further engineering of CDVs. Additionally, the purity assessments confirm that CDVs produced by extrusion are genuine lipid vesicles, not cellular debris or protein aggregates, ensuring the therapeutic value of CDVs as drug delivery vehicles.

## Materials and methods

### The production process of CDVs

The HEK293 cells, provided from Lonza, were cultured in Gibco FreeStyle F17 (Thermo Fisher Scientific, #A1383501) medium supplemented with 4 mM GlutaMAX (Thermo Fisher Scientific, #35050061) and 0.2% Pluronic F68 (Thermo Fisher Scientific, #24040032). The culture was maintained at 37 °C in an 8% CO_2_ atmosphere, with agitation at 120 rpm for a duration of up to 5 days. The cell suspension obtained at the time of harvest was used to generate CDVs, while EVs were isolated from the conditioned medium from the same parental cells. Production of CDVs was performed as described previously^21^. In brief, the cell suspension at 5 × 10^5^ cells/mL was extruded serially through filters with pore sizes of 10, 3, and 0.4 µm (Whatman, USA) using an intermediate-scale extruder ES50 (MDimune Inc, Seoul, Korea). The crude CDVs were subjected to DNA digestion using Benzonase nuclease treatment (Millipore, #70664-3) at 10 U/µg DNA, for 90 min at 37 °C. Next, the suspension was centrifuged for 10 min at 3000×*g*, and the supernatant was collected and processed by tangential flow filtration (TFF) with MidiKros 750 kDa MWCO hollow fiber (Repligen, USA). The TFF-purified CDVs were then filtered through a PES filter with a pore size of 0.45 µm (Sartorious, USA) prior to size exclusion chromatography (SEC) purification using qEV10 column (Izon Science, USA). Finally, CDVs were further concentrated using a 3 kDa Amicon Ultra-15 Centrifugal Filter (Millipore, USA) and stored at − 80 °C before use. For the isolation of EVs, the conditioned medium was centrifuged at 10,000 for 30 min (Beckman Coulter, USA) to remove cellular debris or larger vesicles and subjected to the identical purification process described for CDVs above.

### Proteomics analysis of CDVs

Purified CDVs and EVs together with their parental cells, were analyzed for their proteome profiles. Cell pellets (50 μg) were solubilized in 8 M urea (GE Healthcare, #17-1319-01) and 100 mM Tris–HCl (Invitrogen, #AM9855-G). For CDVs and EVs, a total protein of 50 µg of each sample was precipitated with chilled acetone (Sigma-Aldrich, #179124-500ML) at − 20 °C overnight and solubilized using the same buffer as described for cell pellets. The sample of each group was reduced with 10 mM dl-Dithiothreitol (Sigma-Aldrich, #D0632-10G) at 56 °C for 30 min and alkylated with 25 mM iodoacetamide (Sigma-Aldrich, #I1149-25G) at 25 °C for 30 min. The sample was diluted with 100 mM Tris–HCl to decrease the urea concentration to 1 M, pH 8. The protein mixture was then digested by Trypsin/Lys-C Mix (Promega, #V5073) at 37 °C for 16 h in a ratio of 1:50 (enzyme: protein, w/w). The resultant peptides were cleaned using an OASIS SPE cartridge (Waters, USA), dried in Savant SpeedVac (Thermo Fisher Scientific, USA), and resuspended in 0.1% formic acid (Thermo Fischer Scientific, #LS118-4) in water at 0.5 µg/µL. Digested peptides were analyzed using Q-Exactive plus mass spectrometry with an EASY nLC 1000 liquid chromatography system (Thermo Fisher Scientific, USA). The mass spectrometer was operated in data-dependent mode with a full scan (m/z 350–2000) followed by MS/MS for the top 20 precursor ions in each cycle and the data-dependent neutral loss method. The acquired MS/MS spectra were subjected to searches against the Uniprot-Human database (Jun 2018; 73099 sequences) using SEQUEST software in Proteome Discoverer v2.4 (Thermo Fisher Scientific, version 2.4). Two missed trypsin cleavages were allowed, and the peptide mass tolerances for MS/MS and MS were set to 0.02 Da and 10 ppm, respectively. Other parameters used for the SEQUEST searches included the fixed modification of carbamidomethylation at cysteine (+ 57.021 Da) and the variable modification of oxidation at methionine (+ 15.995 Da). The abundance of each peptide was measured as the number of peptide spectrum match (PSM) events. Data was presented in the form of abundance for each protein.

Proteins identified from all three different replicates of each group were filtered first based on the requirement of at least two peptides per protein. Only proteins with measurable abundance values were included in the analyses. Then, the differentially expressed proteins were identified using the criteria of fold change > 2 and p-value < 0.05 in relative protein abundance values to cells. The most abundant membrane protein markers were selected from the top 5% of the CDV proteome dataset (by abundance) and > seven-fold of the relative abundance compared to the parental cells. Principal component analysis (PCA) and Venn diagram were performed using Proteome Discoverer v2.4.

### Size, polydispersity index (PDI), zeta potential, and particle concentration

The acquisition measurement parameters were previously described^21^. CDVs and EVs were analyzed using the Zetaview instrument (PMX220, Particle Metrix, Meerbusch, Germany) to determine their size distribution, particle number, and zeta potential. Before measurement, samples were diluted in 0.1 µm filtered phosphate-buffered saline (PBS; Gibco, #10010023) to a final volume of 1 mL. For each measurement, the concentrations of samples were measured within the count range of 50–200 particles per frame with two cycles performed by scanning 11 cell positions with the following settings: camera sensitivity: 80; shutter: 100; minimum brightness: 30; temperature: 25 °C. Zeta potential measurements were carried out using samples diluted with DNase, RNase-free water (Invitrogen, #10977015). Polydispersity index (PDI) values were measured using a ZetaSizer 3000-HA (Malvern Instruments, UK). Samples were diluted with PBS to a total volume of 1 mL, and triplicate measurement runs were conducted at 25 °C with a standard refractive index of 1.331.

### Cryo-TEM

The CDVs and EVs at a concentration of 2 × 10^11^ particles/mL were used for cryo-TEM analysis. A total of 5 µL of CDVs or EVs were transferred to a 20 nm-mesh grid. The grids were then subjected to freezing incubation (− 196 °C, 2 h) using Vitrobot (FEI, USA). All samples were observed with TEM (Tecnai G2-F20, FEI, USA). At least 20 images were taken for each group.

### Western blotting

The protein concentration of the sample was first determined using the Pierce BCA Protein Assay Kit (Thermo Fisher Scientific, #23225). Subsequently, 1–2 µg of protein was mixed with 4X Bolt LDS Sample Buffer (Invitrogen, #B0007) and heated at 70 °C for 10 min. The sample was loaded to Bolt 4–12% Bis–Tris Plus Gel (Invitrogen, #NW04120Box) and run at 200 V for 30 min in 1× Bolt MES SDS Running Buffer (Invitrogen, #B0002). Following the electrophoresis, the gel was incubated in 10% ethanol for 30 s and then transferred onto iBlot 2 Transfer Stacks, PVDF membrane (Invitrogen, #IB24001) using iBlot 2 Gel Transfer Device (Invitrogen, USA) according to the manufacturer’s instructions. After blocking the membrane with 5% skim milk in 1× Tris-buffered saline with Tween 20 (TBS-T; Biosensang, #TR2007-100-74) for 1 h, the membrane was probed with primary antibodies, anti-ATP1B3 (Abcam, #ab137055), anti-CD147 (BSG; R&D Systems, #MAB972-100), anti-CD9 (Invitrogen, #10626D), anti-CD63 (Invitrogen, #10628D), anti-CD81 (Abcam, #ab79559), anti-ITGB1 (Abcam, #ab271909), anti-LAMP1 (Sino Biological, #11215-R107), anti-NCSTN (Abcam, #ab247362), and anti-PTGFRN (LS-Bio, #LS-C158765), at 1:2500 ratio overnight. The next day, the membrane was washed three times with 1× TBS-T before being incubated with the secondary antibodies conjugated with horseradish peroxidase, goat anti-mouse (Novus Biological, #NB7539) for CD63, CD81, CD9, and CD147; goat anti-rabbit (Novus Biological, #NB7160) for ITGB1, LAMP1, NCSTN, ATP1B3, and PTGFRN, at 1:2,500 ratio for 1 h. Lastly, the membrane was washed 3 times again with 1X TBS-T and incubated with the ECL Prime Western blotting system (Cytiva, RPN2232) for 30 s and then visualized using ChemiDoc with ECL Prime (Bio-Rad Laboratories, USA).

### Single particle analysis by nanoparticle flow cytometry (nFCM)

#### CDVs marker screening and tetraspanin characterization

Phycoerythrin (PE) or fluorescein isothiocyanate (FITC)-conjugated antibodies were titrated with a wide range of dilutions (1:5–1:10,000) to find the optimal concentration. For each staining reaction, > 2 × 10^8^ particles (purified CDVs or EVs) were incubated with fluorescent primary antibodies for 1 h at 37 °C under shaking, protected from light. After incubation, PBS was used to dilute the sample accordingly and remove the unbound antibodies. Stained samples were then characterized by NanoAnalyzer, nanoparticle flow cytometry (nFCM; NanoFCM Inc, China). The primary antibodies used in the experiments: anti-CD147 (BD Biosciences, #562552), anti-CD 9 (Exbio, #1P-208-T100), anti-CD63 (Exbio, #1P-343-T100), anti-CD81 (Exbio, #1P-558-T100), anti-ITGB1 (R&D Systems, #FAB17783G-100UG), anti-LAMP1 (BD Biosciences, #555801), anti-NCSTN (Novus Biologicals, #NBP2-89825PE), and anti-PTGFRN (Novus Biologicals, #FAB100431P). PE/R-Phycoerythrin Conjugation Kit-Lightning-P-Link (Abcam, #ab102918) was used to conjugate PE fluorophore to the ATP1B3 unconjugated antibody (Novus Biologicals, #H0000483-B01P). Both EVs and CDVs have been characterized against the markers listed above following Lonza’s proprietary method. FlowJo v10 Software was used to analyze the data (BD Biosciences, USA).

#### Membrane and luminal staining of CDVs with dyes

CDVs or EVs at particles of 5 × 10^9^ were incubated with 10 µM of luminal staining dye, carboxyfluorescein succinimidyl ester (CFSE; Thermo Fisher Scientific, #C34554) for 1.5 h at 37 °C with gentle rotation as previously reported by Fortunato et al.^29^. The labeling efficiency of CDVs and EVs was also determined using 1× CellMask Deep Red plasma membrane stain (CMDR; Thermo Fisher Scientific, #C10046) and 1× CellMask Green Plasma membrane stain (CMG; Thermo Fisher Scientific, #C37608) using similar conditions as described for CFSE. For double labeling, the vesicles were stained with 10 µM CFSE followed by 1× CMDR for 1.5 h each at 37 °C with gentle agitation. After incubation, the excess dye was removed by ultrafiltration. Labeled CDVs or EVs were washed with 1× PBS four times before being analyzed by nFCM.

#### Purity assessment of CDVs

The purity of CDVs or EVs was evaluated using a non-ionic surfactant Triton X-100 (Sigma Aldrich, #T9284-100ML). CDVs at 5 × 10^9^ particles were incubated with Triton X-100 at a final concentration of 1% at 4 °C for 1 h. The purity level was determined by comparing the relative particle numbers after the treatment. The particle numbers were normalized with the untreated particles for comparative analysis. A reduction in particle events after adding Triton X-100 indicates the disruption of lipid particles.

## Results

### Production of CDVs and EVs

**Figure 1:**
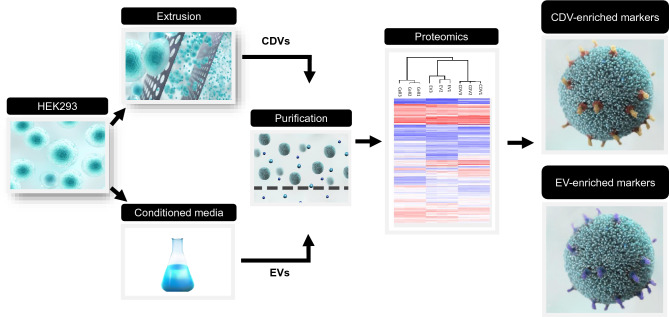
Schematic diagram shows the workflow of the production for CDVs and EVs. CDVs and EVs were produced from HEK293 cells. Cells were passed through membrane filters with different pore sizes using an intermediate-scale extruder, ES50, for CDV production. EVs were isolated from the conditioned medium from the same parental cells. CDVs and EVs were subjected to the same purification methods and then analyzed for proteome profiles. The most abundant CDV protein markers were identified and compared to the EV markers using various analytical methods.

HEK293 cells were chosen for the production of both CDVs and EVs because they are the most suitable cells for diverse cell engineering. Cells were cultured in a chemically defined medium devoid of fetal bovine serum to reduce the contamination of serum-derived proteins or vesicles. First, CDVs were produced using the intermediate-scale extruder, ES50, as previously described^21^. In brief, HEK293 cells were passed through three membrane filters with decreasing pore sizes of 10 µm, 3 µm, and 0.4 µm, three times for each membrane. The post-extrusion mixture was treated with Benzonase nuclease to eliminate the cellular DNAs or other nuclei acids. The mixture was sequentially processed through TFF and SEC to purify CDVs from the residual proteins, nucleic acids, and cellular debris. Similarly, EVs were isolated from the conditioned medium collected for up to 5 days. Importantly, EVs were harvested from the same parental cells and subjected to the identical purification process (TFF and SEC) described for CDVs to minimize the potential variations that could arise from the difference in the purification method. Multiple batches of CDVs or EVs were used for the proteome and particle analyses to identify membrane protein markers abundant in CDVs (Fig. 1).

CDVs were produced using the intermediate-scale extruder, ES50, as previously described. In brief, HEK293 cells were passed through three membrane filters with decreasing pore sizes of 10 µm, 3 µm, and 0.4 µm, three times for each membrane. The post-extrusion mixture was treated with Benzonase nuclease to eliminate the cellular DNAs or other nuclei acids. The mixture was sequentially processed through TFF and SEC to purify CDVs from the residual proteins, nucleic acids, and cellular debris. Similarly, EVs were isolated from the conditioned medium harvested from the same parental cells, HEK293, and subjected to the identical purification process described for CDVs to minimize the potential variations that could arise from the difference in the purification method. Multiple batches of CDVs or EVs were used for the proteome and particle analyses to identify membrane protein markers abundant in CDVs (Fig. 1).

### *C*haracterization of CDVs

**Figure 2:**
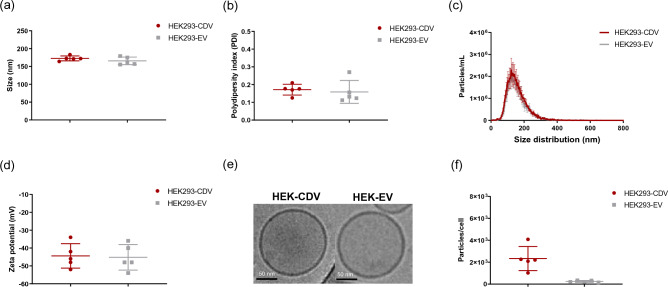
Physical characterization of CDVs. The size (**a**), PDI (**b**), size distribution profiles (**c**), and zeta potential (**d**) were measured from five different batches of CDVs or EVs derived from HEK293. Data represent the mean ± SD. (**e**) Representative high-resolution images are shown from cryo-TEM imaging of HEK293-CDVs and -EVs. Each scale bar indicates 50 nm. (**f**) The productivity was determined from multiple batches of CDVs and EVs from HEK293 using ZetaView and shown as particles produced per cell. Data represent the mean ± SD (N = 5).

We first compared the physical characteristics of CDVs produced from ES50 extruders and EVs secreted naturally from the cells. CDVs and EVs showed a similar size range with a mean size of 172.6 ± 6.9 nm and 165.8 ± 10.7 nm, respectively (Fig. 2a). CDVs also showed no significant difference in polydispersity index (PDI), a measurement of heterogeneity of a particle solution, with PDI of 0.17 ± 0.03 (0.16 ± 0.06 for EVs) (Fig. 2b). We further compared the size distribution of five different batches of CDVs and EVs. Both vesicles showed highly overlapping size distribution profiles (Fig. 2c), with approximately 93.1% of CDVs ranging from 50 to 250 nm (92.2% for EVs) (Supplementary Fig. S1). Overall, CDVs produced from extrusion are highly similar to EVs in particle characteristics. These results are consistent with our previous findings on CDVs and EVs produced from UCMSCs^21^. While a slightly larger size and lower PDI were observed in HEK293-CDVs compared to UCMSC-CDVs, the difference is within experimental variations.

Next, we examined the zeta potential of both vesicles. CDVs and EVs showed a similar zeta potential with − 44.4 ± 6.8 mV and − 45.2 ± 7.2 mV, respectively (Fig. 2d). We then examined the morphology of CDVs and EVs. As revealed by high-resolution cryo-TEM, both vesicles exhibited a round-shaped structure surrounded by a lipid bilayer (Fig. 2e). Subsequently, we compared the productivity between CDVs and EVs. When multiple batches of CDVs and EVs were compared, CDV and EVs yielded approximately 2.3 × 10^3^ ± 1.1 × 10^3^ and 2.5 × 10^2^ ± 8.4 × 10^1^ particles/cell, respectively (Fig. 2f). The productivity of CDVs was significantly higher, approximately 9.4 times, than that of EVs on average, similar to our previous findings in UCMSC-CDVs, 18 times higher than UCMSC-EVs^21^. Together, these results suggest that the extrusion method produces nanovesicles highly similar to EVs in many physical properties but with clear advantages in productivity.

### Proteome analysis and selection of enriched protein markers for CDVs

**Figure 3:**
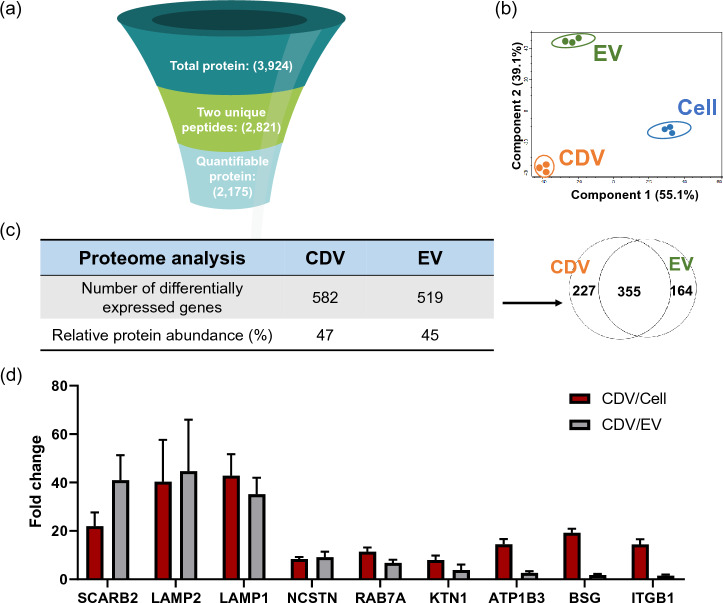
Proteome analyses of HEK-CDVs, -EVs, and their parental cells. (**a**) A diagram illustrates the filtering process of proteome datasets. The total proteins detected (3924) were first filtered based on the criteria of at least two peptides per protein (2821) and further narrowed down to those with measurable abundance values in all groups (2175). The numbers in parentheses represent the total number of proteins in each filtering process. (**b**) Principal component analysis (PCA) was performed using three replicates of each group. The orange, green, and blue dots represent each batch of CDVs, EVs, and cells, respectively. (**c**) Quantifiable proteins were filtered using the criteria of fold change ratio > 2 and p-value < 0.05 relative to cells to identify proteins with differential expression in CDVs and EVs. The total number of differentially expressed proteins was identified for both vesicles (upper row), and the abundance levels were compared to the total abundance of all quantifiable proteins (bottom row). Shared, as well as the unique proteins in CDVs and EVs are presented in the Venn diagram. (**d**) The expression levels of the nine most differentially expressed membrane protein markers of CDVs were compared to cells and EVs using multiple batches of samples. Data represent the mean ± SD (N = 3). *SCARB2* scavenger receptor class B member 2 or lysosome membrane protein 2, *LAMP1* lysosome-associated membrane glycoprotein 1, *LAMP2* lysosome-associated membrane glycoprotein 2, *NCSTN*: nicastrin, *RAB7A* Ras-related protein Rab-7a, *KTN1* kinectin, *ATP1B3* sodium/potassium transporting ATPase subunit alpha-3, *BSG*: basigin, *ITGB1* integrin beta-1.

From the proteome analyses, a total of 3924 proteins were identified from CDVs, EVs, and parental cells. Among these, 2821 proteins contained at least two unique peptides, and the subsequent filtering process identified 2175 proteins quantifiable in all samples with measurable abundance values (Fig. 3a). We compared EV proteome data from this study to the top 100 EV proteins commonly identified in the Exocarta to ensure that our proteome data are coherent with previous findings. Comparative analysis revealed that 86% of the top 100 EV proteins listed in Exocarta were identified in our EV proteome datasets, and 80% of the top 25 EV proteins matched the top 100 EV proteins from Exocarta. (Supplementary Tables S2, S3). This suggests a robust alignment of our proteomics results with the previously published EV studies. Then we conducted a two-dimensional principal component analysis (PCA) to examine how each group segregates based on the protein expression profiles. Each of the CDVs, EVs, and cells displayed distinct proteome signatures, suggesting unique proteome profiles of each group (Fig. 3b). Additionally, three different replicates of CDVs were tightly clustered, indicating a high level of batch consistency of CDVs. We also observed a similar batch consistency from CDVs produced from other cells (data not shown).

Subsequently, we compared the differentially expressed proteins between CDVs and EVs using the criteria of fold change > 2 and p-value < 0.05 in relative protein abundance to cells. More than 500 differentially expressed proteins were identified from CDVs and EVs each. This accounts for nearly half of the total abundance of all quantifiable proteins identified in CDVs and EVs (Fig. 3c). The Venn diagram revealed that 355 proteins were shared between CDVs and EVs, representing 48% of the proteins with differential expression, 227 upregulated in CDVs (30%), and 164 downregulated in CDVs (22%), with respect to EVs. When we further analyzed those up/down-regulated proteins, approximately 77% and 84% of proteins in each sector showed a more than two-fold increase or decrease in protein expression in CDVs, respectively. This shows uniquely enriched protein markers in both vesicles, which is consistent with PCA analysis. Our previous analysis comparing proteome profiles of CDVs and EVs derived from UCMSCs also revealed similarly unique molecular signatures of both vesicles.

We further selected the nine most robust membrane protein markers for CDVs based on the following criteria: top 5% of the CDV proteome dataset by abundance and relative abundance in comparison to parental cells (> seven-fold) (Fig. 3d). Among all these proteins, LAMP1, LAMP2, and SCARB2 were highly abundant in CDVs compared to EVs (> 30-fold increase in CDVs). NCSTN, RAB7A, KTN1, ATP1B3, BSG, and ITGB1 showed a modest increase in CDVs, a fold change of 1.34 to 8.75 compared to EVs. The relative protein abundance values of enriched CDV markers and canonical tetraspanin markers for EVs are shown in Supplementary Table S1.

### Validation of enriched membrane protein markers for CDVs

To further validate the membrane protein markers in CDVs, we used western blotting and nFCM. After considering the abundance levels, subcellular locations, and availability of conjugated antibodies for nFCM analysis, a total of five membrane markers were selected for further validation. Among proteins localized in endo-lysosomal membranes (LAMP1, LAMP2, and SCARB2), only LAMP1 was selected based on its highest abundance value. For proteins expressed primarily on the plasma membrane, NCSTN, ATP1B3, BSG, and ITGB1 were chosen because of the availability of the conjugated antibody for nFCM. RAB7A and KTN1 were not further validated due to the absence of proper reagents. Tetraspanin markers and PTGFRN were included as controls for EVs.

**Figure 4:**
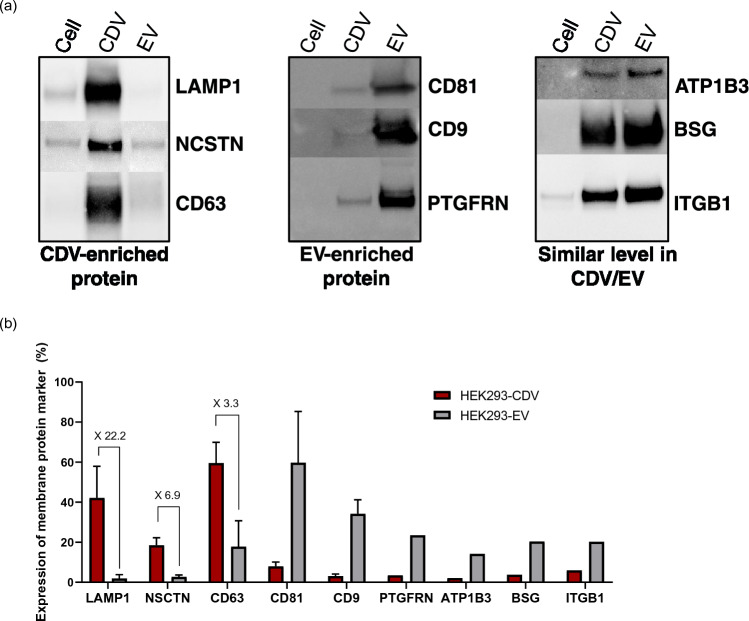
Expression of the nine most enriched CDV membrane protein markers. (**a**) Representative western blotting analyses of membrane proteins that are abundant in CDVs (LAMP1, NCSTN, and CD63), abundant in EVs (CD81, CD9, and PTGFRN), and with similar expression levels between CDVs and EVs (ATP1B3, BSG, and ITGB1). The analyses were determined using an equal amount of total proteins from CDVs, EVs, and parental cells (N = 5). (**b**) nFCM analyses of the identified membrane proteins of CDVs and EV markers. The expression levels of each protein on CDVs were compared to EVs (N = 3 for all except PTGFRN, ATP1B3, BSG, ITGB1). Data represent the mean ± SD.

Western blotting results confirmed that LAMP1 and NCSTN were highly abundant in CDVs compared to EVs or source cells (Fig. 4a). In contrast, a similar expression level of ATP1B3, BSG, and ITGB1 was observed in CDVs and EVs. CD63, one of the tetraspanin markers of EVs, was highly enriched in CDVs, while EVs contained more CD81 and CD9 compared to CDVs. PTGFRN was previously reported as an EV marker^30^ and was also abundant in the EVs in our study. The western blotting results for LAMP1, NCSTN, and tetraspanin markers are coherent with the proteome analyses. Notably, the results of LAMP1 and tetraspanin markers are highly consistent with our previous findings of CDVs from UCMSCs^21^. Similar proteome results were found in the human monocyte cell line, U937, of which LAMP1, NCSTN, and CD63 were also abundant in CDVs (data not shown). These results support that LAMP1, NCSTN, and CD63 are the membrane protein markers that distinguish CDVs from EVs from at least three different cell types. In contrast, no correlation was observed for ATP1B3, BSG, and ITGB1 from both analyses. The discrepancies may be attributed to the modest fold change increase between CDVs and EVs observed in the proteome analyses.

Since western blotting is a semi-quantitative method, we reconfirmed the expression pattern of key membrane proteins using nFCM. Using this method, marker expression can be quantitatively analyzed at a single-particle level. In our experiments, nFCM analysis recapitulated the overall expression pattern of key marker proteins. First, the expression of LAMP1, NCSTN, and CD63 was increased in CDVs compared to EVs. LAMP1 expression was detected in 42.1 ± 15.8% of CDVs, while it was marginal in EVs, 1.9 ± 1.9% (Fig. 4b). CDVs had 18.4 ± 3.8% of NCSTN expression while 2.7 ± 0.9% in EVs. CD63 showed 59.4 ± 10.4% expression in CDVs but 17.7 ± 12.9% in EVs (Fig. 4b). Other tetraspanin markers, such as CD81 and CD9, showed 59.7 ± 25.6% and 34.2 ± 6.9% expression in EVs, whereas CDV showed a much lower level of expression, 7.9 ± 2.1% and 3.0 ± 1.1%, respectively (Fig. 4b). PTGFRN, which is also enriched in EVs, was scarce in CDVs by nFCM. Lower levels of expression were detected for ATP1B3, BSG, and ITGB1 in CDVs compared to EVs, while equal or modest expression of these proteins was observed in western blotting and proteome analyses. The lack of coherent results can be ascribed to the subtle differences in protein expression levels and variations between the analytical methods, such as the sensitivity, detection limits, as well as variability in measurement. Overall, there is a significant alignment between proteome analyses, western blotting, and nFCM for key membrane markers, such as LAMP1, NCSTN, and CD63, for CDVs produced from HEK293 cells.

### Purity assessment of CDVs

**Figure 5:**
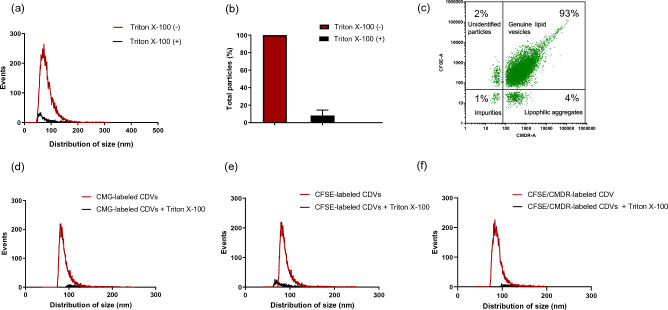
Purity assessment of CDVs using nFCM. (**a**) Representative plot of size distribution following 1% Triton X-100 treatment. CDVs were analyzed before (red) and after (black) Triton X-100 treatment to determine the change in particle number and the size distribution pattern. (**b**) The relative particle number was determined from three different batches of CDVs after the addition of Triton X-100. For comparative analysis, the untreated total particle number was used to normalize the particle number observed upon treatment. Data represent the mean ± SD. (**c**) Representative bivariate dot plot of CDVs double labeled with CFSE and CMDR. In a clockwise direction, the particles in the upper right quadrant represent the genuine lipid vesicles, while the lower right and lower left represent the lipophilic aggregates and other impurities, respectively. The unidentified particles (e.g., impurities interacting with CFSE dye) are located in the upper left quadrant. The number in the plot indicates the percentage of particles in each quadrant. For the purity assessment of fluorescently labeled CDVs, the size distribution was plotted before (red) and after (black) Triton X-100 treatment. The representative plots show the effect of Triton X-100 on the size distribution of CMG-, CFSE-, and double-labeled CDVs (**d**–**f**).

Next, we aimed to examine whether CDVs are bona fide lipid vesicles by disrupting the lipid membrane vesicle using 1% Triton X-100, followed by particle analysis using nFCM. We found that the particle count of CDVs was significantly reduced after treatment with Triton X-100 (Fig. 5a). This result was further verified by using different batches of CDVs, with more than 91.8 ± 6.3% of CDVs successfully digested using Triton X-100 (Fig. 5b). This data strongly suggests that the majority of CDV particles produced by extrusion are genuine lipid vesicles rather than other cellular debris or by-products such as protein aggregates.

Subsequently, we confirmed this by labeling CDVs with fluorescent dyes targeting either the lipid membrane or luminal compartments. CMG or CMDR is a lipophilic membrane dye that labels the CDV lipid membrane, whereas CFSE is a membrane-permeable luminal dye that stains the lumen of CDVs. EVs were employed as a control group. The nFCM analyses showed that 95% and 97% of the CDVs were successfully labeled with CFSE and CMDR, respectively (Fig. 5c). Notably, 93% of the CDVs were labeled with both CFSE and CMDR dyes (Fig. 5c). When multiple batches of samples were used, 93.1 ± 1.6% and 92.5 ± 1.0% of CDVs were positive for CMG and CFSE, respectively, compared to 91.1 ± 1.1% and 89.9 ± 5.1% of EVs (Supplementary Fig. S2a). When comparing co-labeling efficiency, both CDVs and EVs exhibited comparable, high levels of labeled vesicles with CFSE and CMDR, with 90.3 ± 1.1% for CDVs and 90.5 ± 0.77% for EVs (Supplementary Fig. S2a), indicating that CDVs and EVs similarly interact with these dyes. To eliminate any potential labeling artifacts, we disrupted the lipid membrane structure of fluorescently labeled CDVs using 1% Triton X-100. After exposing CDVs to Triton X-100, particle count was remarkably reduced in CMG, CFSE, and CFSE and CMDR double-labeled populations (Fig. 5d–f). When we repeated this in three different batches of CDVs, 92.4 ± 3.5% (CMG) and 90.4 ± 1.9% (CFSE) of CDVs were digested by Triton X-100 on average (Supplementary Fig. S2b). Similar results were observed from co-labeled CDVs and EVs, 92.1 ± 1.5% for CDVs and 90.7 ± 6.9% for EVs (Supplementary Fig. S2c). Taken together, these data confirm that CDVs produced by extrusion are intact membrane-bound lipid vesicles with high purity.

## Discussion

The characterization of CDVs was performed by comparing them to naturally secreted EVs. The results showed that CDVs and EVs had similar size distribution, polydispersity index, zeta potential, and morphology. We have again confirmed that HEK293-CDVs produced from extrusion have similar physical characteristics to HEK293-EVs but with higher yield. Previously, we have reported that UCMSC-CDVs have been found to preserve many of the biological effects of EVs while offering distinct advantages in productivity, making CDVs more attractive for large-scale production^21^. Therefore, we have provided evidence from multiple cell sources that our proprietary extrusion technology is a highly efficient process to produce lipid nanovesicles.

We also conducted a series of assays to confirm that CDVs are genuine lipid vesicles, similar to the naturally secreted EVs. nFCM was used to characterize CDVs and EVs at a single particle level. In fact, it was previously reported that nFCM is a more reliable platform than nanoparticle tracking analysis (NTA) while providing both quantitative and qualitative analyses of vesicles^31–33^. The purity of CDVs was assessed by disrupting the lipid membrane structure with Triton X-100, as it is widely described as a standard method for accurate lipid membrane vesicle quantification or purity assessment^34–36^. The results showed that more than 90% of CDVs are bona fide lipid vesicles. Triton X-100-resistant particles are considered to be protein aggregates and other impurities. We also verified this result using lipophilic and luminal dyes that can stain vesicles. To overcome concerns regarding the potential miscounting of the aggregates of lipophilic dye as vesicles, we also assessed co-labeling efficiency between dyes targeting the membrane (CMDR) and luminal (CFSE) compartments. As expected, a high percentage, > 90%, of the double-labeled population was observed from CDVs. It is speculated that the CMDR-positive vesicles lacking the luminal dyes (CFSE) may be the lipophilic dye aggregates or membrane fractions. The identity of CFSE-positive particles lacking CMDR remains unresolved, and these particles are likely the protein impurities that interacted with the CFSE dye outside CDVs (Fig. 5c). Small fraction of impurities, possibly protein and cellular debris that cannot be stained by either CFSE or CMDR were detected as double-negative particles. Notably, highly similar results were obtained between CDVs and EVs, suggesting that extrusion can produce nanovesicles with a comparable purity as EVs. The co-labeling approach might be further used to distinguish impurities or aggregates from CDVs and enable the quantification of intact lipid vesicles. This method can be adapted to quality control CDVs in various manufacturing steps, therefore ensuring the reliability and consistency in the quality of the CDVs for various applications.

Finally, we characterized unique surface protein markers of CDVs in comparison with EVs. As previously reported, CDVs formed by the extrusion of cells are expected to be generated from more diverse lipid sources such as plasma and subcellular organelle membranes and therefore exhibit slightly different marker patterns from EVs^21,37^. To obtain more precise knowledge of CDV membrane protein markers, we combined the results of three distinct analytical methods: proteome analysis, western blotting, and nFCM. The results from the analyses of CDVs reflect the differences in their origin or mechanism of production compared to EVs by showing distinct enrichment of membrane proteins between these two vesicles. Consistent with our previous study on UCMSC-CDVs, we provide evidence that CDVs produced from HEK293 are also enriched in both plasma membrane proteins and subcellular organelle membrane proteins, whereas EVs contain relatively lower levels of organelle membrane proteins. Additionally, the differential expressions of well-known tetraspanin markers in both vesicles support that CDVs possess a distinct membrane composition from EVs. The diverse source of membrane proteins may elucidate a possible mechanism underlying the higher yield of CDVs than naturally secreted EVs. Studies from Nasiri Kenari (2019) and Zhang (2022) support the findings of this study by showing distinct proteome composition between CDVs and EVs^38,39^. Our previous cellular uptake study of UCMSC-CDVs showed enhanced cellular uptake of CDVs, compared to EVs, which are believed to be mediated by CD63 and LAMP1^21^. Therefore, this finding may suggest the universal role of specific membrane markers, such as CD63 and LAMP1, in the cellular uptake of CDVs.

While gaining insights into the abundant CDV markers for HEK293, it is necessary to investigate if CDVs from different cell sources possess the same set of markers, as each cell type may present distinct markers. With the knowledge of CDV-enriched membrane proteins, CDVs can be further engineered for targeting capabilities, cargo delivery, and enhanced therapeutic potential. Besides the well-described advantage in yield, the excellent purity level of CDVs makes them a suitable candidate for industrial-scale production and efficient drug delivery platforms.

## Conclusions

We have demonstrated that CDVs are genuine membrane-bound lipid vesicles, rather than protein and other impurities, with a characteristic surface marker profile. With proven productivity, this study reaffirms the potential of this vesicle-based technology for use in therapeutic applications and provides the basis for the development of CDV engineering and companion diagnostic strategies using the identified CDV-specific biomarkers.

### Supplementary Information

Supplementary Information.

## Data Availability

The datasets generated during and/or analyzed during the current study are available from the corresponding author upon reasonable request.
