# Supplementary material for: Unraveling the surface marker signature of cell-derived vesicles via proteome analysis and nanoparticle flow cytometry
Source: Sci Rep. 2024 Jan 2;14:121. doi: 10.1038/s41598-023-50279-x (PMC10762029; doi:10.1038/s41598-023-50279-x)
Supplement: Supplementary file 1 — Supplementary Information. [file 41598_2023_50279_MOESM1_ESM.pdf]

Unraveling the surface marker signature of cell-derived vesicles via proteome analysis and nanoparticle flow cytometry

Hui-Chong Lau[1], Ilaria Passalacqua[2], Jik-Han Jung[1], Yerim Kwon[1], Davide Zocco[2], Sung-Soo Park[1], and Seung Wook Oh[1,3,*]

[1] BioDrone Research Institute, MDimune Inc., Seoul, Korea

[2] Lonza Siena., Siena, Italy

[3] BioDrone Therapeutics Inc., Seattle, USA

[*] Correspondence should be addressed to swoh@mdimune.com

Supplementary Table S1. The nine most differentially expressed membrane protein markers in CDVs.

No	Protein Name	Gene name	CDV (Abundance)	EV (Abundance)	CDV/EV	CDV/Cell	EV/Cell
1	Lysosome-associated membrane glycoprotein 1	*LAMP1*	625,753,223	18,317,643	34.16	41.69	1.22
2	Lysosome-associated membrane glycoprotein 2	*LAMP2*	618,379,380	15,638,189	39.54	36.38	0.92
3	Basigin	*BSG/CD147*	578,831,342	356,519,809	1.62	19.18	11.81
4	Kinectin	*KTN1*	502,081,461	179,452910	2.79	7.82	2.81
5	Integrin beta-1	*ITGB1*	407,439,475	304,557,675	1.34	14.28	10.68
6	Ras-related protein Rab-7a	*RAB7A*	387,167,648	59,032,236	6.56	11.42	1.74
7	Sodium/potassium-transporting ATPase subunit beta-3	*ATP1B3*	368,836,860	150,996,699	2.44	14.48	5.93
8	Nicastrin	*NCSTN*	333,029,841	38,070,235	8.75	8.28	0.95
9	Lysosome membrane protein 2	*SCARB2*	325,954,252	8,133,431	40.1	21.41	0.53
10	Tetraspanin CD63	*CD63*	274,787,551	49,466,431	5.55	29.24	5.26
11	Tetraspanin CD81	*CD81*	131,259,149	525,212,037	0.25	16.10	64.44
12	Tetraspanin CD9	*CD9*	94,886,099	257,505,787	0.37	5.04	13.67
13	Prostaglandin F2 receptor negative regulator	*PTGFRN*	381,966,809	1136,219,822	0.34	3.94	11.73

The respective protein and gene names are based on the accession ID. The measurable abundance values of the membrane protein markers in CDVs (fourth column) were compared to those in EVs (fifth column). The relative abundance levels of CDV membrane proteins were compared to both EVs and parental cells. The protein abundance values of EVs relative to cells were also included in the analysis. Tetraspanin markers and PTGFRN were used as controls.

Supplementary Table S2. Comparison of the top 100 EV proteome entries from Exocarta with the EV proteome dataset (current study)

Rank (Exocarta)	Protein name (ExoCarta)	Rank (current study)	Rank (Exocarta)	Protein name (ExoCarta)	Rank (current study)	Rank (Exocarta)	Protein name (ExoCarta)	Rank (current study)	Rank (Exocarta)	Protein name (ExoCarta)	Rank (current study)
1	CD9	109	26	VCP	236	51	HSPA1A	-	76	HIST2H4A	-
2	PDCD6IP	43	27	TPI1	107	52	GNAI2	267	77	GNB1	37
3	HSPA8	23	28	PPIA	105	53	ANXA1	402	78	THBS1	-
4	GAPDH	-	29	MSN	59	54	RHOA	123	79	RAN	-
5	ACTB	4	30	CFL1	242	55	MFGE8	6	80	RAB5A	1406
6	ANXA2	142	31	PRDX1	106	56	PRDX2	298	81	PTGFRN	12
7	CD63	509	32	PFN1	172	57	GDI2	133	82	CCT5	21
8	SDCBP	34	33	RAP1B	145	58	EHD4	595	83	CCT3	22
9	ENO1	7	34	ITGB1	91	59	ACTN4	214	84	AHCY	96
10	HSP90AA1	5	35	HSPA5	421	60	YWHAB	311	85	UBA1	69
11	TSG101	629	36	SLC3A2	154	61	RAB7A	448	86	RAB5B	1144
12	PKM	17	37	HIST1H4A	-	62	LDHB	90	87	RAB1A	231
13	LDHA	101	38	GNB2	240	63	GNAS	157	88	LAMP2	999
14	EEF1A1	9	39	ATP1A1	13	64	RAB5C	355	89	ITGA6	380
15	YWHAZ	84	40	YWHAQ	100	65	ARF1	261	90	HIST1H4B	-
16	PGK1	70	41	FLOT1	326	66	ANXA6	162	91	BSG	75
17	EEF2	38	42	FLNA	19	67	ANXA11	651	92	YWHAH	541
18	ALDOA	225	43	CLIC1	149	68	ACTG1	-	93	TUBA1A	-
19	HSP90AB1	2	44	CCT2	18	69	KPNB1	141	94	TKT	906
20	ANXA5	608	45	CDC42	251	70	EZR	86	95	TCP1	25
21	FASN	8	46	YWHAG	710	71	ANXA4	723	96	STOM	-
22	YWHAE	40	47	A2M	-	72	ACLY	72	97	SLC16A1	274
23	CLTC	11	48	TUBA1B	10	73	TUBA1C	665	98	RAB8A	547
24	CD81	47	49	RAC1	148	74	TFRC	876	99	MYH9	203
25	ALB	-	50	LGALS3BP	-	75	RAB14	1050	100	MVP	-

Supplementary Table S3. Top 25 EV proteins (current study) vs Exocarta proteome entries

Rank (Current study)	Protein name (current study)	Rank (ExoCarta)
1	VCAN	-
2	HSPA1B	-
3	LGALS3BP	50
4	ACTB	5
5	HSP90AA1	10
6	MFGE8	55
7	ENO1	9
8	FASN	21
9	EEF1A1	14
10	TUBA1B	48
11	CLTC	23
12	PTGFRN	81
13	ATP1A1	39
14	CLU	-
15	HSP90AB1	19
16	CCT8	-
17	PKM	12
18	CCT2	44
19	FLNA	42
20	BASP1	-
21	CCT5	82
22	CCT3	83
23	HSPA8	3
24	GAPDH	4
25	TCP1	95

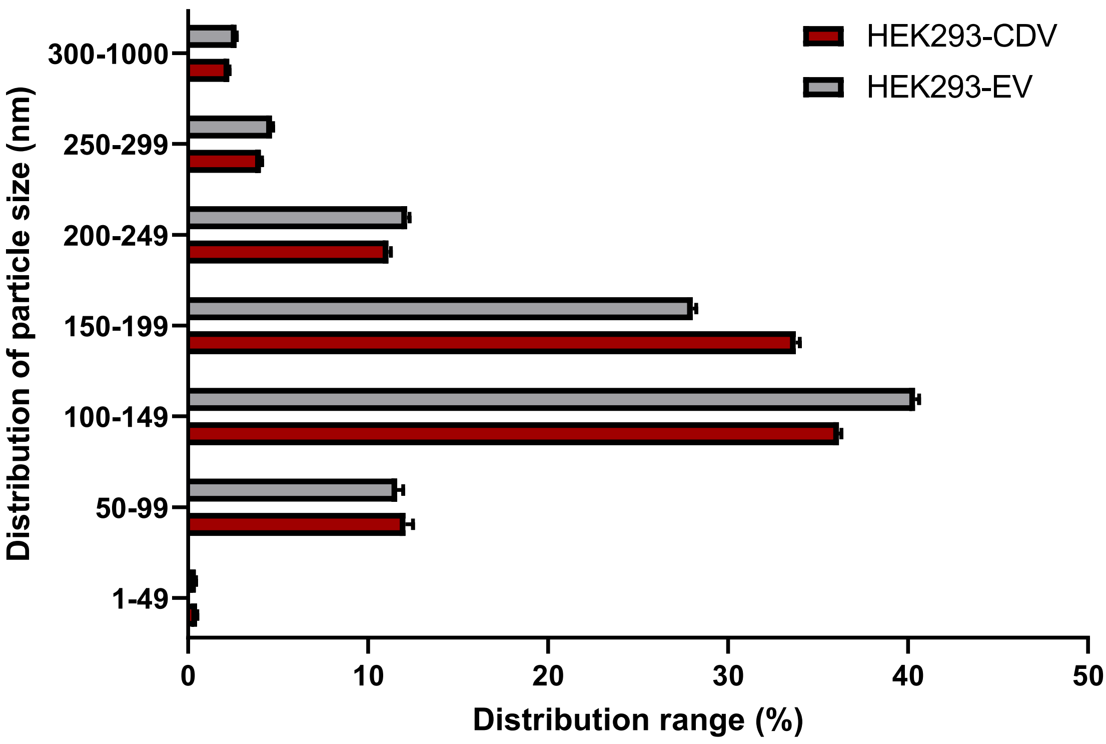

Supplementary Figure S1. Size distribution of CDVs and EVs. Data represent the mean ± SD (N=5).

(a)

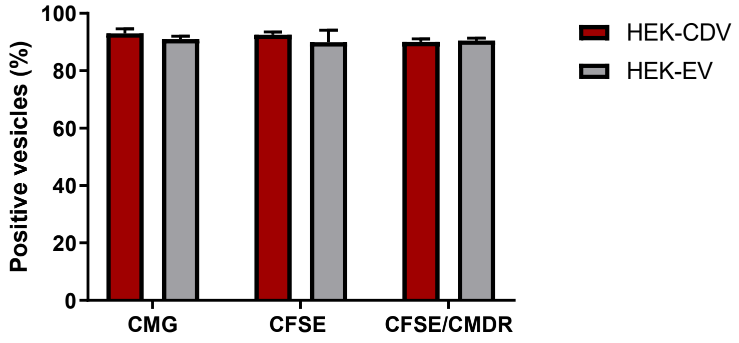

(b)

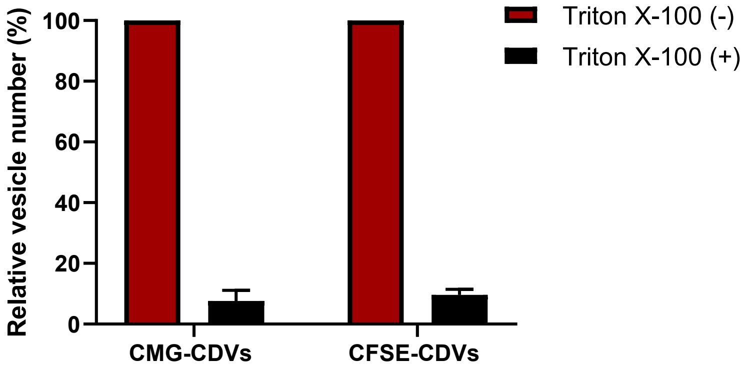

(c)

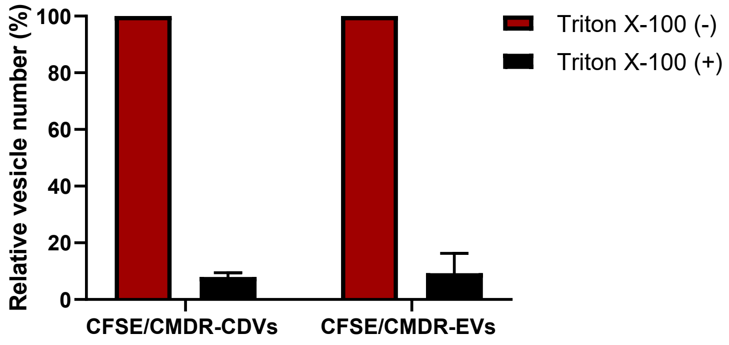

Supplementary Figure S2. (a) Membrane and luminal staining CDVs. CDVs were labeled with single (CMG or CFSE) or double (CFSE and CMDR) fluorescent dyes. The labeled CDVs were then analyzed by nFCM. The positive vesicles indicate the fluorescent vesicles in the population. EVs were used as the control group. Data represent the mean ± SD (N=3). (**b-c**) Purity assessment of CDVs using nFCM. The relative particle number of CDVs was determined after Triton X-100 treatment. CDVs labeled with (**b**) single fluorescent dye, (CMG or CFSE), and (**c**) both different fluorescent dyes (CFSE and CMDR) were analyzed before (red) and after (black) addition of Triton X-100. EVs were used as the control group for double labeling. The untreated CDVs were used to normalize each data for comparative analysis. Data represent the mean ± SD (N=3).
